# Hepatitis E and Potential Public Health Implications from a One-Health Perspective: Special Focus on the European Wild Boar (*Sus scrofa*)

**DOI:** 10.3390/pathogens13100840

**Published:** 2024-09-27

**Authors:** Fabio Castagna, Giovanna Liguori, Renato Lombardi, Roberto Bava, Anna Costagliola, Antonio Giordano, Massimiliano Quintiliani, Denise Giacomini, Francesco Albergo, Andrea Gigliotti, Carmine Lupia, Carlotta Ceniti, Bruno Tilocca, Ernesto Palma, Paola Roncada, Domenico Britti

**Affiliations:** 1 Department of Health Sciences, University of Catanzaro Magna Græcia, 88100 Catanzaro, Italy; fabiocastagna@unicz.it (F.C.); tilocca@unicz.it (B.T.); palma@unicz.it (E.P.); roncada@unicz.it (P.R.); britti@unicz.it (D.B.); 2 Mediterranean Ethnobotanical Conservatory, 88054 Catanzaro, Italy; studiolupiacarmine@libero.it; 3 Local Health Authority, ASL, 71121 Foggia, Italy; giovanna.liguori@aslfg.it (G.L.); renato.lombardi@aslfg.it (R.L.); 4 Department of Veterinary Medicine and Animal Productions, University of Napoli Federico II, 80100 Naples, Italy; anna.costagliola@unina.it; 5 Sbarro Institute for Cancer Research and Molecular Medicine, Center for Biotechnology, Department of Biology, College of Science and Technology, Temple University, 1900 N 12th Street, Philadelphia, PA 19122, USA; antonio.giordano@temple.edu; 6 Department of Medical Biotechnology, University of Siena, 10100 Siena, Italy; 7 Sbarro Health Research Organization ETS, 10060 Candiolo, Italy; mquintiliani@unite.it; 8 Ministry of Health, 00144 Rome, Italy; d.giacomini@sanita.it; 9 Department of Management, Finance and Technology, University LUM Giuseppe Degennaro, 70100 Casamassima, Italy; albergo@lum.it; 10 Interregional Park of Sasso Simone and Simoncello, 61021 Carpegna, Italy; ambiente@parcosimone.it; 11 ASL Napoli 3 SUD, Department of Prevention, 80053 Castellammare di Stabia, Italy; carlottaceniti@gmail.com

**Keywords:** hepatitis E, zoonosis, one health, wild boar, public health

## Abstract

The hepatitis E virus (HEV) has become increasingly important in recent years in terms of risk for public health, as the main causative agent of acute viral hepatitis. It is a foodborne disease transmitted to humans through the consumption of contaminated water or contaminated food. Human-to-human transmission is sporadic and is linked to transfusions or transplants. The main reservoirs of the hepatitis E virus are domestic pigs and wild boars, although, compared to pigs, wild boars represent a lesser source of risk since their population is smaller and the consumption of derived products is more limited. These peculiarities often make the role of the wild boar reservoir in the spread of the disease underestimated. As a public health problem that involves several animal species and humans, the management of the disease requires an interdisciplinary approach, and the concept of “One Health” must be addressed. In this direction, the present review intends to analyze viral hepatitis E, with a particular focus on wild boar. For this purpose, literature data have been collected from different scientific search engines: PubMed, MEDLINE, and Google scholar, and several keywords such as “HEV epidemiology”, “Extrahepatic manifestations of Hepatitis E”, and “HEV infection control measures”, among others, have been used. In the first part, the manuscript provides general information on the disease, such as epidemiology, transmission methods, clinical manifestations and implications on public health. In the second part, it addresses in more detail the role of wild boar as a reservoir and the implications related to the virus epidemiology. The document will be useful to all those who intend to analyze this infectious disease from a “One-Health” perspective.

## 1. Introduction

The hepatitis E virus (HEV), named for its “Enteric” transmission route and its ability to cause “Epidemics” [1], is the most common cause of acute hepatitis and jaundice worldwide, and may have zoonotic origins.

Hepatitis E is usually benign and self-limiting, but it can become chronic and fatal in immunocompromised individuals, especially in pregnant women in developing nations [2]. The virus has infected nearly one-third of the global population and is the leading cause of acute viral hepatitis globally, elevating it to an emerging public health concern [3]. Although the epidemiological landscape varies across countries based on their level of development, hepatitis E is found in both developed and developing countries [4]. Initially, it was considered a travel-related virus in Western countries, linked to endemic regions. However, in recent years an increasing number of autochthonous cases have been reported in European countries, many of which are linked to the consumption of undercooked meat or inadequately cured pork [5].

In industrialized nations, the primary sources of hepatitis E infection are contact with animals and the consumption of raw or undercooked meat, particularly liver from pigs, wild boar, and deer [6]. In contrast, poor hygiene and sanitation remain significant contributors to hepatitis E transmission in developing countries. Notably, the first epidemiological investigation of hepatitis E, conducted in India in the early 1950s, traced the outbreak to sewage pollution in the Yamuna River [7]. There are eight known genotypes of HEV, with genotypes 1, 2, 3, and 4 being the most common in human infections. Genotypes 3 and 4 are zoonotic, with the domestic pig serving as a principal animal reservoir [8].

Besides humans, various domestic and wild animal species have been shown to carry HEV or HEV-related viruses [9], complicating efforts to control the disease and contributing to its persistence. Among the hepatitis viruses, HEV stands out as the only one with foodborne zoonotic transmission and non-primate animal reservoirs [10]. Pig farms are known to have high rates of HEV infections, and the consumption of pork products—especially those containing pig liver—is associated with these infections [11]. HEV is also found in wild boars, and exposure to HEV is linked to the consumption of game meat or hunting activities [12,13]. Interactions between domestic and wild suids may play a role in the maintenance and spread of HEV in both animal populations [14]. The function of viruses as foodborne pathogens has been brought to the attention of the worldwide scientific community by several pieces of research. When it comes to managing foodborne microbiological contamination, preventive approaches are often ineffective when viruses are involved [15]. In 2017, 5079 cases of foodborne disease were reported in Europe, and it has been shown that viruses play a major role as the causal agents [16]. According to the EFSA Advisory Committee, the primary viral agents associated with foodborne disease outbreaks are norovirus and hepatitis A and E viruses [17]. Although hepatitis E is not as widespread as other foodborne diseases, concern about it has been growing within the EU. The hepatitis E virus (HEV), a leading cause of acute viral hepatitis, has become an increasing threat to public health in recent years. According to the World Health Organization (WHO), three million people develop acute hepatitis and 20 million contract HEV each year [18]. To understand the virus’s impact on global health systems, it is worth noting that complications related to hepatitis E claim the lives of over 57,000 individuals annually [19]. Over the past 10 years, more than 21,000 clinical cases and 28 fatalities related to this infection have been reported in Europe, reflecting a ten-fold increase in incidence during this period [20]. This review article on HEV will focus on genotypes, prevention and management strategies, diagnostic techniques, and current knowledge gaps. Special attention will be paid to wild boar and its epidemiological significance as an animal reservoir.

## 2. Methods

Literature data were collected from the PubMed, Google Scholar, and MEDLINE databases. Several search terms related to the manuscript’s topic were used, including “HEV epidemiology”, “Extrahepatic manifestations of Hepatitis E”, “HEV infection control measures”, and “Hepatitis E and wild boar”, among others. The use of “AND” and “OR” operators depended on how the search terms were combined. Only research articles published in English were included in the review process. Two tables summarizing the most significant content of the text have been created.

## 3. HEV Virology, Genome, and Classification

The classification of the virus is continuously evolving due to the isolation of new strains in various animal species (e.g., mammals, birds, and fishes) [21]. HEV belongs to the family Hepeviridae. Members of the family are assigned to two subfamilies, five genera, and ten species. Members of the subfamily Orthohepevirinae infect mammals and birds; the hepatitis virus belongs to genus *Paslahepevirus* [21,22]. It is a small, non-enveloped particle with an icosahedral capsid, measuring 27–34 nm in diameter. HEV is a single-stranded, positive-sense RNA virus [23]. The viral genome is approximately 7.2 kb in length, comprising a short 5′ noncoding region (NCR), a 3′ NCR, and three open reading frames (ORF1-3). The genome is flanked by a 7-methylguanosine cap at the 5′ end and a polyadenine tail at the 3′ end [24].

ORF1, located at the 5′ end, encodes non-structural polyproteins essential for viral replication. ORF1 includes several functional domains that resemble helicases, methyltransferases, papain-like cysteine proteases, and RNA-dependent RNA polymerases found in other positive-sense RNA viruses [25]. Recently, an overlapping reading frame within ORF1, known as ORF4, has been identified. ORF4 is crucial for the proper function of HEV RNA polymerase and is activated during endoplasmic reticulum stress [26].

The bicistronic subgenomic RNA system translates ORF2 and ORF3, which overlap [27]. ORF2 encodes the viral capsid protein, located at the 3′ end of the genome. This capsid protein contains three potentialglycosylation sites and a signal peptide sequence. Mutations in the glycosylation sites can prevent the virus from forming infectious particles. Leucine residues at positions 477 and 613 in the capsid protein are critical for forming neutralizing epitopes. Two epitopes between amino acid residues 578 and 607 can be partially recognized by neutralizing monoclonal antibodies targeting the capsid protein [28].

ORF3 encodes a small phosphoprotein associated with the cytoskeleton. The N-terminus of ORF3 interacts with HEV RNA, helping it combine with the capsid protein. The multifunctional C-terminus of ORF3 plays a role in the pathogenesis and morphogenesis of virion formation. The ORF3 protein, present on the surface of released HEV particles, is responsible for virion egress from infected cells. Although the production of ORF3 protein is not necessary for viral replication, assembly, or in vitro infection, it is necessary for viral infectivity in vivo. Depending on the genotype, ORF3 encodes a phosphoprotein of 113 or 114 amino acids [29].

While ORF3 is necessary for infection in macaques, it is not required for viral replication, assembly, or infection in the hepatoma cell line in vitro [30].

This protein is essential for viral egress from infected cells. HEV particles in serum are associated with lipids and the ORF3 protein, allowing HEV to circulate in the blood in a quasi-enveloped form. However, HEV is excreted in stools as non-enveloped virions [31].

HEV can exist as either a quasi-enveloped virus, where the capsid is coated with an exosomal membrane, or as a non-enveloped virus, where capsid proteins interact with cellular receptors to facilitate viral entry and replication. The non-enveloped form is ten times more infectious than the quasi-enveloped form, though both forms are capable of causing infection [32].

The C-terminal region of ORF2 has been observed to bind the HSC70 protein, a member of the heat shock protein family, on the cell surface, facilitating viral entry [33]. Heparan sulfate proteoglycans (HSPGs) have also been identified as cell surface receptors [27].

In summary, ORF 1 encodes the non-structural proteins involved in RNA replication, including RNA helicase and RNA-dependent RNA polymerase [34]. ORF2 encodes structural proteins essential for forming viral capsids, which have antigenic regions that elicit immune responses, making them promising candidates for vaccine development [35].

ORF3, which is necessary for viral survival, replication, and egress from host cells, is only found in blood-borne, enveloped HEV particles [35].

## 4. HEV Epidemiology

The *Hepeviridae* family, which includes HEV, is divided into two primary subfamilies: *Orthohepevirinae*, comprising four genera, and *Piscihepevirus*, which belongs to the *Parahepevirinae* subfamily. This classification is based on a study published by the International Committee on Taxonomy of Viruses (ICTV) in 2022 [22,36]. Within the *Orthohepevirinae* subfamily, the genera *Paslahepevirus* and *Rocahepevirus* are capable of infecting both domestic and wild animals, humans, and bats, while *Avihepevirus* affects only birds [30]. The *Paslahepevirus* genus contains two species: *P. balayani* and *P. alci* (specific to moose). *P. balayani*, formerly known as *Orthohepevirus A*, infects humans and a variety of animals and has eight distinct genotypes. Genotypes 1–4 are particularly dangerous to humans, with genotypes 1 and 2 responsible for large outbreaks of HEV, while zoonotic infections from genotypes 3 and 4 typically cause sporadic or clustered cases in developing nations. [37,38].

The HEV-3 genotype also includes a distinct variant, *HEV-3ra*, which infects rabbits but has a closely related human strain [39]. HEV genotypes 5 and 6 have only been detected in wild boars in Japan [40].

HEV-7, first identified in dromedary camels, was later found in a liver transplant patient suffering from chronic hepatitis [41]. HEV-3 is widely distributed among pigs in various geographic regions, including the Americas, Europe, Africa, Japan, Southeast Asia, and Oceania, while HEV-4 is predominantly found in pigs from China, Japan, and Indonesia [41].

The distinct features of HEV, particularly its genotype, result in different clinical and epidemiological profiles depending on the site of infection. Of the four HEV genotypes that cause illness in humans, the clinical and epidemiological characteristics differ between developed and developing nations. In regions where HEV is endemic—genotype 1 in Asia and Africa, genotype 2 in Mexico and West Africa, and genotype 4 in Taiwan and China—hepatitis E manifests in two primary ways: as isolated cases in developed countries (genotype 3) or as severe outbreaks and sporadic cases in endemic regions. It is estimated that approximately one-third of the global population has been exposed to HEV, though the exact burden remains unclear. Approximately 3 million people show symptoms each year, with 20 million contracting the virus and at least 600,000 deaths attributed to HEV annually [42,43].

Hepatitis E is more prevalent in low-income countries with limited access to clean water and poor sanitation. Major hepatitis E outbreaks have been reported in Asia, the Middle East, Africa, and Central America [44].

People living in overcrowded temporary shelters or refugee camps, particularly after natural disasters, may be especially vulnerable. Socioeconomic and ecological factors play a significant role in shaping global epidemiological patterns. In developing countries, large-scale outbreaks often result from contaminated water or direct human contact [45]. In contrast, hepatitis E was previously considered a travel-associated illness in the West. However, in recent years, an increasing number of autochthonous cases have been identified in European countries. It is now recognized that HEV is endemic within the European Union, and that among the seven genotypes known to infect both animals (HEV-6) and humans (HEV-1 to -4 and HEV-7), most autochthonous HEV-3 infections in EU countries are linked to the consumption of undercooked or inadequately cured pork [1].

In low- and middle-income nations, the illness is linked to the consumption of contaminated water and/or poor sanitation and hygiene. The illness manifests in these regions as both occasional instances and outbreaks. Epidemics can affect hundreds to thousands of individuals and typically occur after periods of fecal pollution of drinking water sources [46].

Several of these outbreaks happened in places where there is a humanitarian crisis or ongoing warfare, such war zones, as well as in camps for refugees or other displaced people, where access to clean water and sanitation is particularly difficult [47,48].

The majority of cases in these regions are brought on by genotype 1 viral infection, with genotype 2 virus infections occurring far less frequently [49].

The hepatitis E illness is uncommon in locations with improved water supply and sanitation. In these context, the cases are normally ascribed to genotype 3 of the virus, which is mostly transmitted by eating undercooked animal flesh (including animal liver, especially pig). Water or other food contamination is not a factor in these instances [50,51], while cases due to transfusion or organ transplantation have become more common [52].

The incidence of hepatitis E increased in Europe between 2005 and 2015, with a total of 21,081 cases reported [53]. According to data published by the ECDC (European Center for Disease Prevention and Control), the number of confirmed cases rose steadily each year, representing a tenfold increase from 514 cases in 2005 to 5617 in 2015 [54]. In this same decade, 28 deaths associated with the infection were reported across five countries. It should be noted, however, that the true burden of human disease/infection due to hepatitis E in Europe remains unclear. This is due to varying levels of awareness, testing, and surveillance activities, as well as the lack of published information in many European Union and European Economic Area member countries [55].

In Italy, the unique SEIEVA system (Integrated Epidemiological System of Acute Viral Hepatitis) is used to track hepatitis E cases. Since 2007, data on anti-HEV IgM positive have been gathered within this system [56].

Hepatitis E accounts for 332 (or 2%) of the almost 16,000 instances of acute hepatitis reported to SEIEVA since 2007. An analysis of the annual case numbers indicates a rising trend, largely driven by factors other than travel to endemic regions, which has remained steady since the start of monitoring. One factor contributing to the spread is the growth in wild boar reservoir populations.

The population of wild boars in Europe and Italy has increased significantly in recent years [57]. This impressive demographic growth is substantially due to historical factors, including the abandonment of many formerly cultivated hilly and mountainous areas, leading to the expansion of wooded areas, as well as uncoordinated reintroduction programs. While some of these factors have brought notable benefits to biodiversity, the resulting increase in potential prey has aided the conservation of species at risk of extinction [58].

Localized analyzes indicate that wild boar populations have also increased significantly in the rest of Europe over the last 30 years. This growth has substantially contributed to negative interactions between wild boar and humans, or between wild and domestic animals, thereby increasing the spread of diseases. As a reservoir for several important zoonotic diseases, wild boars pose risks to both human and animal health [59]. Concurrently, this demographic increase has led to a rise in the consumption of game in Italy, amplifying the risk of disease transmission. Consequently, the hygiene and safety of game meat plays a fundamental role in safeguarding public health [60]. Contact with at-risk species and the consumption of their raw or undercooked meat represent a potential danger for the transmission of important zoonoses, including the hepatitis E virus (HEV) [61]. Among wildlife, however, the wild boar is the natural reserve of HEV and, therefore, health surveillance in this species is fundamental to reducing the risk of transmission to humans and domestic animals [62]. Evidence of wild boar-to-domestic pig transmission dates back to the end of the 1990s, when a study in Australia demonstrated that 17% of pigs raised in semi-wild conditions tested positive for anti-HEV antibodies.

It was not until 2004 that the virus was first detected in wild boar in Japan. In subsequent years, the virus was isolated in wild boars in Europe, including France, Germany, Hungary, Italy, the Netherlands, Belgium, Sweden, Portugal, Estonia, and Spain [63].

In several regions of Italy, cases positive for anti-HEV antibodies have been reported recurrently. The data presented so far should prompt reflection on the complex epidemiology surrounding this disease [62,64,65,66,67,68,69]. Therefore, two different epidemiological patterns can be observed, which are schematized in Table 1.

## 5. Transmission Routes

**Figure 1:**
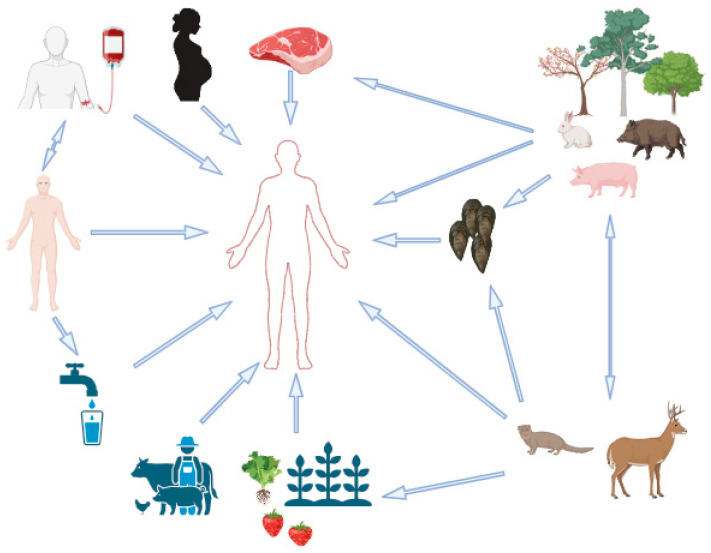
Transmission routes of HEV.

The majority of HEV transmissions, which pose a serious threat to public health, occur through the consumption of raw or undercooked meat from infected animals (zoonotic, foodborne transmission) and by drinking fecally contaminated water in highly endemic areas with inadequate sanitation practices (waterborne transmission). In rare cases of acute hepatitis E, the exact mechanism of HEV transmission remains debated, and the origins of the viral infection can be unclear. In such cases, additional factors such as living arrangements, hygienic conditions, and population immune level may also be connected to the illness. Three further pathways of HEV transmission are thought to exist, according to recent studies: parenteral (blood-borne), human-to-human, and vertical (mother-to-child) transmission (perinatal transmission). Although these pathways of transmission are assumed to be less common, the increasing body of research supporting this notion supports preventative actions that can lower the risk of HEV infection [70,71,72] (Figure 1).

A probable method of transmission is contact with diseased animals [73]. This also explains how slaughterhouses, pig farmers, veterinarians, hunters, and forestry workers have a greater seroprevalence compared to the general population.

Interactions between wild and domestic suids could aid in the dissemination and maintenance of HEV in both repositories [41]. In Figure 1 are summarized the possible routes of HEV transmission.

### 5.1. HEV in Domestic Pigs and Wild Boars

Domestic pigs and wild boars are the primary reservoirs of the hepatitis E virus. Because of their smaller number, and less consumption of related products, wild boars provide a lesser danger than pigs.

Pigs are mostly asymptomatic when infected, and the length of viremia and the rate at which it spreads via feces are determined by variables related to both animal care and business management. The virus is partially present in meat and may be spread by excrement and bile. According to research [74], less than 10% of pigs arrive at the butcher viremic. This phenomenon might indicate a potential danger of cross-infection with meat contamination during the stages of slaughter, evisceration, and processing.

In every nation where the investigation was conducted, pigs with HEV infections were mostly discovered in slaughterhouses that were connected to farms. Additionally, it has been shown that the incidence of HEV varies greatly across farms, production methods, and nations; non-industrial agricultural systems have been shown to be particularly vulnerable in some investigations [75,76].

Over the last 20 years, a significant body of data has been collected suggesting that wild pigs also represent a significant reservoir of HEV. Since wild boars (*Sus scrofa*) and domestic pigs (*Sus scrofa domesticus*) are closely related, it is not unexpected that they are equally vulnerable to HEV. The discovery of HEV antibodies in wild-caught pigs in Australia at the end of the 1990s is what prompted the initial report indicating that HEV may also infect wild boars [77]. A few years later, in Japan, the first partially sequenced HEV isolate from a wild pig was discovered [78]. A tight relationship between this strain and HEV-3 isolates previously discovered in Japanese patients and farm pigs was shown by comparing their ORF2 partial sequences (298 nucleotides) [78]. A few months later, the first complete genome sequences of HEVs found in wild boars were published. It was determined that this strain is part of HEV-3 and that it has 99.7% nucleotide similarity with other isolates from deer and humans when compared to entire or nearly complete HEV isolates [79]. As of right now, HEV-3 makes up the majority of HEV strains found in wild boars. In Japan’s wild boar population, several HEV-4 variations have also been identified [80,81]. Furthermore, the HEV isolates that have exclusively been found in wild boars—JBOAR135-Shiz09, wbJOY_06, and wbJNN_13—have been described. Two new genotypes, HEV-5 (JBOAR135-Shiz09) and HEV-6 (wbJOY_06 and wbJNN_13), have been attributed to these HEV strains. Less than 80% of the nucleotide identity between these two genotypes and HEV-1 to -4 is shared [80,81]. It has been proposed that the wbJNN_13 and wbJOY_06 isolates correspond to two different subtypes within the HEV-6 genotype [82] and that their whole genomes are 80.4% identical [80]. These two genotypes have not yet been linked to any human cases; hence, it is unknown if they may spread to humans. There are very little data on the clinical symptoms that HEV causes in wild boar.

In one study, there was no difference in the biometric characteristics (body length and weight) of wild boars with HEV infection and those without it [83].

In another case, a male viremic wild boar did not show any clinical signs and tested negative for anti-HEV IgG [78]. Increased levels of bile acids (BA), alanine aminotransferase (ALT), and gamma-glutamyl transferase (γGT) were observed in conjunction with mild diarrhea and reduced feed intake in 3-month-old wild boars that were experimentally infected with the wild boar HEV-3 strain, either intravenously or through contact. Additionally, a case of moderate lymphoplasmacytic hepatitis was reported. The feces of infected wild boars contained larger virus loads of HEV than the feces of miniature pigs infected under the same conditions [84].

HEV RNA was also detected in the spleen, small and large intestines, liver, and gall bladder of the infected wild boars. Additionally, persistent infections in two wild boars with naturally occurring HEV-3 have been reported [85].

These two animals exhibited significant titres of anti-HEV antibodies in their blood, but for 12–16 weeks their feces showed signs of viremia and/or viral shedding. There were no histological lesions or clinical signs of hepatitis in the chronically infected wild boars, and no HEV RNA was found in their livers or other organs [85].

Numerous investigations have been conducted, mostly in Europe and Japan, to ascertain the frequency of HEV RNA and/or antibodies in wild boars. Japan and Europe had seroprevalences ranging from 1.6 to 41.6% and 4.9 to 57.4%, respectively. There have also been reports of RNA prevalences as high as 10.3% in Japan and as high as 68.2% in Europe. These findings unequivocally demonstrate that wild boars in Europe and Japan are often infected with HEV, and that wild pigs in these regions most likely serve as a reservoir for HEV. HEV prevalence may be impacted by several variables, including the sample year [80,86,87,88], the geographic area [64,86,89,90,91,92,93], the population of wild boars [92,94,95], and the management circumstances [86,96].

Adults and subadults often have a greater HEV seroprevalence than juveniles [14,65,86,87,89,97]. There was no discernible difference between wild boars that were male or female [13,65,81,83,86,95,98,99]. The presence of the virus in wild boars exposes domestic pigs to infection, because they often encounter wild boars, other wild animals, hunters, and those who consume wild boar meat.

### 5.2. HEV Transfer between Domesticated and Wild Suid Animals

Research has shown that HEV can overcome the barrier that separates farmed pigs from wild boars. HEV excretion in the feces, seroconversion, and viremia may result after oral and intravenous inoculation of wild boar HEV strains to domestic or miniature pigs [84,100]. Wild boars infected with HEV can transmit the virus to domestic or miniature pigs through contact, particularly in areas where domestic pigs are raised near wildlife [84,85]. The fecal–oral transmission is facilitated by the proximity of domestic and wild swine. This is common globally, notably in regions with high HEV seroprevalence like Tuscany and Corsica [65,87]. Hybrid pigs, resulting from the breeding of wild boars and domestic pigs, play a significant role in HEV epidemiology. These hybrids, which exhibit both wild and domestic behaviors, are more likely to interact with domestic pigs and form larger groups. In Corsica, 43.5% of hybrid pigs had anti-HEV antibodies, a higher seroprevalence compared to domestic pigs (88%) and pure wild boars (26%) [87]. Then, hybrid pigs could act as a middleman in the HEV transmission chain between domestic and wild pigs. Based on whole/nearly complete [95,101] or partial [83,87,96,102,103,104] sequences, many investigations have shown that pig and wild boar strains of HEV-3 and HEV-4 have 90–98% similarity. For instance, a comparison of full-length sequences revealed 96.9% similarity between the sequences of a Mongolian pig strain and a wild boar HEV-3 strain found in Germany [95]. A domestic pig HEV-3 strain and a wild boar strain that were both identified in Corsica have been shown to have a partial ORF2 sequence homology of 97.5% [105]. Based on partial ORF2 sequences, it was demonstrated that two wild boar HEV-3 strains obtained in Hungary had more similarities with pig strains (97–98% identity) [104].

Additionally, a molecular evolutionary research has suggested that a subtype of HEV-3 became endemic in Japan following the importation of infected pigs from Europe in the 1960s, where it was then transferred from pigs to wild boars [106].

The findings suggest dynamic HEV-3 and HEV-4 transmission between farmed pigs and wild boars. Proving direct transmission requires identifying nearly identical strains in both groups. The potential for HEV-5 and HEV-6 spread remains unclear, as these genotypes have only been found in wild boars with no recorded experimental transmission to pigs. Investigating the impact of cross-infection on HEV dynamics, the role of wild boars as reservoirs, and the possible transmission to humans and other animals, is essential. Additional research should explore transmission routes via surface water and environmental contamination and determine if HEV transfers between pigs and deer [79,88].

### 5.3. HEV in Foods Derived from Suidae

The majority of research indicates detection rates between 2 and 8%; however, records for pig liver detection rates range from 0 to 21%. Using quantitative analysis, HEV genome quantities ranging from 20 to 10^7^ RNA copies/g are reported. There are studies on wild boar liver from Europe and Japan that provide 2–38% detection rates. The HEV genome’s amounts varied from 40 to 10^8^ RNA copies/g.

It is essential to consider that wild boars and pigs are very sensitive to carrying the HEV genome, and that a significant portion of the viral genome may be present throughout these animals’ lifetimes.

Investigations have detected HEV RNA in meat and muscle samples with varying rates: 0–6% in pig muscle, 0–12% in wild boar muscle. Quantitative data show HEV genomes in wild boar muscles at 500–4000 copies/g. HEV RNA may be present in up to 12% of muscle samples. Studies also reveal high detection rates (16–47%) of HEV in pig-derived meat products, especially sausages and liver [107,108]. The utilization of livers from many animals for sausage manufacture and the ensuing mixing effect account mostly for the reported detection rates, which are greater than those found for pig livers. The liver-containing products had between 4 and 2 × 10^6^ copies of the HEV genome per gram [109].

Additionally, HEV RNA was detected at more variable detection rates, ranging from 0 to 20%, in sausages that did not include liver as well as in sausages whose liver content was unknown [110]. The presence of an infectious virus is not always indicated by the detection of HEV RNA. To prove the infectivity of food products that have previously tested positive for HEV RNA, significant efforts have been undertaken, but since there are no effective cell culture models for HEV propagation, measuring HEV infectivity is challenging [111]. As a result, the number of studies examining the presence of infectious HEV in animal organs or meat products meant for human consumption is limited. In Japan, pig liver sold at retail was found to contain HEV, which was effectively shown to be infectious using an A549 cell culture system [112]. HEV was isolated from pig liver sausages purchased at retail locations in France using a 3D cell culture model, proving the existence of an infectious virus [113]. Experimental inoculation of pigs with sample homogenates proved the presence of infectious HEV in commercially available pig livers [114,115,116]. Case reports provide more convincing evidence that eating food derived from animals may spread the HEV virus. Seven people in France were involved in a hepatitis E epidemic, according to Colson et al., after consuming figatelli, a kind of liver sausage [72]. Similar HEV sequences were found in the local sausage samples and patients. A related example involving the identification of identical HEV sequences in a French patient with hepatitis E and the residual figatelli has subsequently been reported [117]. In a separate incident on a coastal French island, three individuals had hepatitis E [118]. Epidemiological investigations identified hog meat as the source of HEV infection in an immunocompromised patient. Identical HEV sequences were found in the piglet, the patient’s samples, and a liquid manure sample from the farm where the piglet was born [119]. By proving that the patient’s and the meat sample’s HEV sequences were similar, two case reports from Japan determined that grilled wild boar meat was the cause of the illness [120]. Similarly, it was discovered that the HEV sequences in a wild boar liver matched those in two Japanese patients who had consumed the liver [121].

### 5.4. HEV Exposure by Contact with Suidae

HEV transmission through contact with pigs or wild boars is widely hypothesized and supported by serological studies comparing those with occupational animal exposure—such as veterinarians, butchers, and pig farmers—to controls. These exposed groups show higher HEV-specific antibody prevalence. However, the direct comparison of studies is challenging due to different assays and other variables, such as dietary habits. Some research has found no significant seroprevalence differences between exposed and non-exposed individuals [122]. Nonetheless, HEV transmission by this channel seems to be widespread, since most investigations show greater prevalences in people who had contact with pigs. Numerous pieces of serological research with hunters and forest workers have been carried out to investigate the route by which HEV is transmitted from wild animals to people. Higher HEV-specific antibody prevalences were identified in the forest workers’ groups in two investigations conducted in Germany and France [123,124] as compared to the control groups. According to a piece of Japanese research, the seroprevalence of wild boar hunters was much greater (25.3%) than that of a group of locals (5.5%) from the same region [125]. Similar research was carried out in Germany, and the results showed that hunters had a somewhat higher HEV seroprevalence than the German community as a whole [126]. A detailed analysis revealed that hunters who used gloves while disemboweling wild boars had significantly fewer anti-HEV antibodies compared to those who did not use gloves. This suggests that HEV transmission is likely through contact with wild animals, particularly wild boars. Although evidence of disease from direct contact is rare, other studies have linked antibody presence to viral transfer from pigs or wild boars. Notably, a French patient with acute hepatitis E frequently interacted with a pet pig and its excretions, highlighting a potential transmission route [127].

Comparisons of HEV strains from a patient and a pig revealed nucleotide sequence identities of 92–98%, indicating a close relationship between the strains, though not identical. This suggests the patient likely contracted a unique HEV quasispecies from the pig through direct contact or excretions. Additionally, HEV transmission has been observed in surgeons training on pigs and through contact with contaminated organs, confirming hepatitis E as an occupational infection acquired from handling infected pig organs.

## 6. Clinical Manifestations of HEV Infections

### 6.1. Animals

Animals can contract HEV at various stages of their growth, with most studies indicating that pigs become infected between 8 and 15 weeks of age [96,128,129,130], and some arrive positive at slaughter [131].

According to research, between 2 and 15% of pigs have HEV when they are killed. HEV viremia usually lasts for two weeks, during which time the virus excretes through feces in weeks three through seven [129].

Pigs with the virus have tiny liver lesions, yet they do not show any symptoms at all. Pigs develop hepatitis E antibody seroconversion around weeks 11 and 13, as the mother’s antibodies begin to diminish. IgM anti-HEV antibodies peak first, and then IgG anti-HEV antibodies [130].

The dynamics of the illness in wild boar are comparable to those in domestic pigs, with the likelihood of viral chronification and infection frequency rising with age [78].

In pigs, the liver is thought to be the main organ targeted by HEV replication. Although modest hepatic lesions suggestive of hepatitis have been reported in both naturally infected and experimentally infected pigs, HEV infection in pigs is usually asymptomatic. Even though the liver plays an important part in HEV pathogenesis, several elements are still unknown, such as the processes behind liver damage and the liver’s function in viral reactivation [132].

Numerous studies have assessed histological results connected to HEV infection in pigs and wild boar. Experimental models using the intravenous injection of HEV resulted in histological observations that were in line with acute hepatitis. These comprised expanded portal tracts, localized parenchymal necrosis, acidophilic masses, inflated hepatocytes, and significant inflammatory infiltrates in the lobules. However, pigs infected by contact only showed mild to moderate multifocal lymphoplasmacytic hepatitis and single-cell necrosis of hepatocytes. A quick and broad viral diffusion occurs in intravenous infections, whereas in natural contact infections HEV may stay longer in hepatic tissue; this is most likely the cause of the discrepancies in liver histology results between the two infection models (intravenously injected and contact-infected).

The majority of viral antigen was found in liver sinusoidal endothelial cells and Kupffer cells, two types of cell that present antigen in the sinusoidal vascular region. The star-shaped form of Kupffer cells, which are resident tissue macrophages in the liver, and their placement next to or inside the endothelium lining of hepatic sinusoids help to identify them [132].

Pigs are reservoirs for HEV, and people may acquire the disease from other pigs if they encounter the excrement of sick pigs. The lack of clinical illness in infected pigs, at least with the strains examined, may make swine an unsuitable animal model for human HEV; however, the swine model is still helpful in comprehending some elements of HEV pathophysiology and epidemiology [132].

### 6.2. Humans

HEV incubates for three to eight weeks post-exposure. Most acute HEV infections are asymptomatic or minimally symptomatic, but 5–30% can develop acute icteric hepatitis, starting with fever, body pains, nausea, vomiting, and malaise. This progresses to an icteric phase with dark urine and jaundice lasting about a week. Symptoms then resolve in the convalescent phase. HEV genotypes 1 and 2 cause more severe acute hepatitis, while genotypes 3 and 4 can lead to acute-on-chronic liver failure in individuals with chronic liver disorders. Immunity develops after acute infection, though reinfection may still occur with a lower risk of symptomatic hepatitis [133,134].

Immunocompetent individuals often do not require special care for acute HEV infections. However, HEV1 in pregnant women, particularly in the third trimester, can cause severe complications like liver failure and has a high maternal mortality rate [135]. Research by Gouilly et al. [136] found that HEV1 proliferates more efficiently than HEV3 in placental cells, leading to increased cell death and necrosis at the maternal–fetal interface.

HEV1 increases viral loads and tissue damage by producing more pro-inflammatory cytokines and virions. During pregnancy, a Th2-dominated immune response replaces Th1, protecting the fetus, but this can impair the monocyte–macrophage system. Hormonal changes, including elevated progesterone and estrogen, further worsen outcomes by enhancing HEV replication, particularly in the third trimester [137,138].

It is crucial to conduct research to comprehend how HEV variations and immunological, hormonal, and genetic factors influence the severity of HEV1 and HEV2 in pregnancy. Acute hepatic encephalopathy (ALCLF) can have up to 70% fatality, marked by rapid liver function decline and severe comorbidities. HEV may trigger ACLF, often misdiagnosed as drug-induced liver disease.

An American study found HEV to be the cause of some cases misdiagnosed as drug-induced liver damage, using the European Association for the Study of the Liver’s ACLF criteria. While most acute HEV infections are mild or asymptomatic, pregnant women and those with chronic liver disease are at higher risk. The infection’s severity varies with HEV genotype, necessitating further research to fully understand and mitigate these risks. The symptoms of a hepatitis E virus (HEV) infection can vary, ranging from asymptomatic to severe acute and fulminant hepatitis. Hematological, neurological, and renal diseases are only a few of the extrahepatic consequences that can result from HEV infection in addition to these basic liver signs. The discovery of HEV in human placenta, breast milk, urine, and neural cells suggests that the pathophysiology of these extrahepatic manifestations may entail direct viral replication in these organs or systemic immune responses in non-hepatic tissues [138]. This underscores the potential severity of HEV infections and the need for vigilance.

Neurological manifestations are common in HEV infections, with 16.5% of HEV patients in France showing symptoms. Reported worldwide, these include Bell’s palsy, encephalitis, myelitis, neuralgic amyotrophy, and Guillain-Barré syndrome. Guillain-Barré is characterized by rapid paralysis from autoimmune nerve damage, with anti-HEV IgM often present. In the Netherlands, Belgium, and France, 5–8% of cases involve this condition [137].

In the Netherlands and the UK, neuralgic amyotrophy has been reported in up to 10% of cases. It is characterized by painful upper extremity mononeuropathy and motor weakness, frequently affecting the brachial plexus. While encephalitis and myelitis are less common symptoms, both illnesses demonstrate a strong correlation between HEV infection and certain neurological problems.

The hematological symptoms of HEV infection include thrombocytopenia, autoimmune hemolytic anemia, and glucose-6-phosphate dehydrogenase (G6PD) insufficiency. G6PD insufficiency occurs in 70% of acute cases, while 23% present with autoimmune hemolytic anemia. Thrombocytopenia may result from anti-platelet antibodies, hypersplenism, reduced hepatic thrombopoietin, or bone marrow suppression. Less common issues include pancytopenia, cryoglobulinemia, and lymphoproliferative diseases. Treating HEV infections can resolve cryoglobulinemia, which is found in both acute and chronic cases. Cryoglobulinemia, IgA nephropathy, and membranoproliferative glomerulonephritis (MPGN) are examples of renal manifestations. Acute HEV genotype 3 infections are the main cause of MPGN and IgA nephropathy, which have a somewhat decreased glomerular filtration rate. With the right care, cryoglobulinemia, which affects both acute and chronic HEV patients and is particularly common in recipients of solid-organ transplants, also goes away [138].

First reported in 2018, chronic HEV infection is more likely among immunocompromised people who are unable to rid themselves of the virus on their own, such as those with HIV, hematologic diseases, or solid-organ transplants. A chronic infection is indicated by HEV RNA that persists for more than three months. Most frequently, genotypes 3 and 4 are implicated. Although sofosbuvir, pegylated interferon-alpha, and ribavirin are among the treatment alternatives investigated, the pathophysiology of chronic infection remains incompletely understood. Research indicates that some immune responses and compromised T-cell responses may be factors in chronic infections [133].

Approximately 20% of solid-organ transplant patients develop persistent HEV infection, often asymptomatic or with minor liver enzyme increases. Accurate diagnosis typically requires HEV RNA detection since elevated liver enzymes are common in post-transplant issues. Careful treatment is needed to distinguish chronic HEV infection from other post-transplant diseases that may cause graft hepatitis. Recent studies underscore the complexity and impact of HEV in immunocompromised or transplant patients.

In a study of liver transplant recipients, 1.45% of the group had chronic hepatitis E. HEV IgG seroprevalence rates were 29% and 28% before and after transplant, respectively, and there were two cases of HEV re-infection documented. HEV-infected liver transplant recipients had a higher chance of developing cirrhosis.

Pischke et al. found that 1.6% of de novo chronic HEV cases were caused by HEV RNA and IgG testing among 493 participants. The study highlights the need for HEV RNA testing in patients with elevated liver enzymes when no other cause is evident.

Thirteen had hepatitis caused by other viruses, seven had graft hepatitis, and thirty-three patients experienced graft rejection among the seventy patients who had increased liver enzymes. The study found that 1.6% of cases of de novo chronic HEV infection were caused by HEV RNA and anti-HEV IgG testing. The research emphasized that, unless there is an obvious other cause of hepatitis, HEV RNA testing is necessary in individuals with increased liver enzymes. Five patients (four who had received kidney transplants and one who had received a heart transplant) had chronic infection, and all cases were of HEV genotype 3. Foodborne transmission was suspected, especially from undercooked deer, boar, and pig. Chronic HEV infection in kidney transplant recipients can cause liver disease to advance quickly, eventually leading to decompensated liver failure soon after acute infection [133,139].

Patients with hematologic malignancies, including non-Hodgkin’s lymphoma and acute lymphoblastic leukemia, have also been reported to have HEV infection. It has been seen that immunosuppressive medication can cause chronic viremia and reactivate HEV post-stem-cell transplantation. Among these immunocompromised groups, chronic HEV infection is a serious problem that calls for caution and specialized care techniques [140].

Twenty-one individuals from internal medicine and rheumatology clinics with anti-HEV IgG positive were examined in a retrospective multi-center European case study. People with rheumatoid arthritis, psoriatic arthritis, systemic lupus erythematosus, and primary immunodeficiency were among the cohort’s members. Of the patients, 24% continued to have HEV infections for more than six months, and 33.3% acquired chronic infections that lasted longer than three months. HEV genotypes 3c and 3f were present in four of these individuals, but genotype 1 was likely imported in one.

The patients’ ages varied from 29 to 75 years, and their underlying medical conditions included rheumatoid arthritis, retroperitoneal fibrosis, and common variable immunodeficiency. Five individuals received treatment with ribavirin; one patient had rheumatoid arthritis and maintained viremia for 16 weeks after ending Abatacept-induced immunosuppression. This study demonstrates that individuals with rheumatologic illnesses, as well as those undergoing transplantation and HIV, are susceptible to persistent HEV infection. However, no instances of persistent HEV infection were discovered in a different research of 488 individuals with inflammatory bowel disease receiving immunomodulator treatment, indicating that routine HEV screening may not be required for these patients. Henoch–Schönlein purpura, myasthenia gravis, myocarditis, myositis, thyroiditis, and other extrahepatic symptoms of HEV infection have all been documented. However, more investigation is needed to prove beyond reasonable doubt that these illnesses and HEV infection are related [133].

## 7. Measures of Prevention of HEV Infections

There are gaps in knowledge concerning the virus that need to be filled in order to implement effective counteractions. To implement an effective prevention program, it is necessary to understand how the virus is distributed in reservoir animal populations, humans, and food products that can carry the infection. Paying attention to the possible sources that can generate infection and the methods of transmission, it is noted that not much is known about the transfer from pigs to people, and more research is required in order to understand the transmission in both humans and animals. Furthermore, it is unclear how much foodborne transmission occurs and what role animals other than pigs play in zoonotic transmission to humans. There are also gaps in knowledge on how long HEV may survive in the environment, especially on surfaces that come into contact with food, as well as how effective removal techniques are. While awaiting the filling of these knowledge gaps, observance of simple rules can help in the prevention of infection.

The most common oral and fecal routes of transmission for hepatitis E include drinking contaminated water and eating undercooked pork or wild pig. Hygienic behaviors can help lower the risk of infection. Boiling or frying the HEV virus can render it inactive; five minutes are required to achieve inactivation of the virus at a temperature above 90 °C, while reaching a temperature of 71 °C maintained for 20 min at the core is required for inactivation in meals [141].

HEV is susceptible to the chlorine disinfection that needs to be used on sawdust, water sources, and pesticides [46,142,143].

Stem cells, solid-organ transplantation (SOT), transfusions of blood and blood products, and items generated from pigs can all be sources of infection, particularly in immunocompromised patients. By screening biological samples in blood banks, infection occurrence can be prevented [144,145].

In Europe, the main reservoirs for HEV-3 are pigs and wild boars, although the virus has been detected in other animals like deer and rabbits [41,146].

Large-scale farming, the lack of a depopulation time period, the absence of a quarantine period, and interaction with other domestic animals are risk factors linked to higher HEV prevalence in pigs [147]. After introduction, HEV can persist on farms for multiple years [148,149]. To reduces the burden to public health, risk mitigation for HEV infection should occur at the slaughterhouse, as well as at the farm level. Effective control strategies for HEV in pig farms are little known [150].

A control approach that is universally dependable must be established due to the high frequency of HEV in European swine farms. HEV infections might be avoided by using external biosecurity measures, including quarantine zones and routine testing of cleaning protocols using bacterial growth assays [151]. Carrying out mass vaccination of susceptible individuals and animals [143] would also limit the spread of the infection. Table 2 summarizes possible preventive measures that can be applied at three different levels, namely, on exposure routes, on specific categories of people, and on infected people.

## 8. Diagnosis of HEV Infection

In HEV surveillance investigations, the primary diagnostic technique employed is the enzyme-linked immunosorbent test. Anti-HEV antibodies may be found by ELISA in a variety of materials, including meat juice, body cavity transudate, and serum. Samples such as feces, liver, serum, muscle, semen, and food items can all contain HEV RNA, which can be found using reverse transcriptase-polymerase chain reaction methods [152,153].

Since HEV is only temporarily detectable in serum, the most commonly used samples for virus detection are urine, liver, and particularly feces. Jothikumar et al. (2006) created a popular RT-PCR approach that is well-known for its excellent sensitivity and specificity. More extensive research is required to draw firm results, even if RT-qPCR is appropriate for HEV detection in hepatic transudate. Due to financial and technological constraints, large-scale field prevalence investigations may not always be possible using RT-qPCR.

It might be challenging to obtain appropriate serum samples, particularly from wild boars, because of the interval between death and sampling. When properly diluted (1:10), liver transudate offers pigs and wild boars a useful substitute for serum in the ELISA test for anti-HEV antibodies. In postmortem investigations for diagnosis, monitoring, or study where blood collection is impracticable, this matrix is very helpful [154].

In humans, following HEV exposure, the incubation period ranges from 15 to 60 days [37]. Three weeks after infection, detectable HEV RNA appears in the blood and stool, just before the development of clinical symptoms and subsequent rise of biochemical markers. Four weeks after infection, immunoglobulin M antibodies usually surface, and a few days later, IgG antibodies. IgG antibodies are detectable for years following primary HEV infection, but IgM antibodies only last for three to four months [133].

HEV RNA is detected by molecular testing, which generally uses tests based on nucleic acid amplification techniques to determine active infection. To identify and amplify RNA, these tests use reverse transcription polymerase chain reaction, focusing on conserved areas of ORF2 and ORF3 to identify all four HEV genotypes. Although polymorphisms can result in false negative results, adding small changes to the groove binding increases sensitivity and reduces this danger. When HEV RNA is consistently detected for three months or more, it is indicative of chronic hepatitis E, which is typically seen in immunocompromised people.

Because of the possibility of undetectable antibody levels, molecular testing is preferable over antibody testing in these individuals. It is essential to track the HEV RNA viral load to evaluate treatment outcomes. A viral load that is undetectable indicates that therapy is working, but levels that are persistent or rising suggest that treatment is not working and that other measures are required. In immunocompromised patients, viral RNA recurrence indicates relapse. Anti-HEV antibodies can be used to diagnose acute HEV infection. IgM antibodies start to show up four weeks or less after the initial beginning of symptoms, but IgG antibodies can be found a few days after IgM and last for years. For serological testing, enzyme immunoassays are employed in conjunction with HEV NAAT. Serological testing is not without limits, even if the inclusion of anti-HEV IgA testing is said to boost sensitivity, but in individuals with reduced antibody production, such as those under long-term immunosuppression, it is unreliable [155].

Furthermore, there is cross-reactivity between the Epstein–Barr virus and CMV in antibody testing. Enzyme immunoassays for HEV antigen detection are useful in both acute and chronic situations. On the other hand, positive rates are less than those of RT-PCR. Antigen detection in feces does not necessarily indicate the existence of infectious HEV virions, even if it may continue to be positive for a few weeks after HEV RNA becomes undetectable. The existence of infectious HEV virions is not directly correlated with the results of enzyme immunoassays for HEV antigen detection in feces, even though these results can be high for weeks after V RNA becomes undetectable [156].

Certain features should be looked at since it is vital to distinguish between acute HEV infection and acute autoimmune hepatitis (AIH). Smooth muscle antibodies were found in both circumstances at comparable frequencies and titers, which is in line with earlier research. On the other hand, only acute AIH subjects had antinuclear antibodies. Furthermore, it was not possible to reliably distinguish between the two forms of acute hepatitis using liver histology. Notably, the simplified score and IgG levels were able to distinguish between acute AIH and acute hepatitis E [157].

## 9. Treatments for HEV Infections

Currently, there are no authorized therapies for HEV infection.

Most acute HEV infections resolve on their own and do not require special care. However, severe hepatitis and liver failure can result from acute HEV infection; these cases mostly afflict people with chronic liver disorders and pregnant women. Acute and severe primary or secondary HEV hepatitis is the preferred indication for antiviral medication ribavirin therapy. By reducing guanosine triphosphate pools, which in turn inhibits inosine monophosphate dehydrogenase and prevents HEV RNA replication, it appears to suppress HEV replication [158].

Due to its teratogenic potential, contrasting findings on the administration of ribavirin to expectant mothers with severe hepatitis caused by HEV-1 or HEV-2 or liver failure have been recorded. However, in the final three months of treatment with Ribavirin after organogenesis had finished, no teratogenic consequences were noted in pregnant women [159]. Ribavirin is also used in liver, renal, pancreas, heart and lung transplant recipients and in patients undergoing hemodialysis for the treatment of chronic HEV infections (HEV3 or HEV4) [160], who need a reduced immunosuppression treatment and cannot be treated with pegylated interferon-alpha, the latter usually being used as a first-line therapeutic approach in solid-organ transplant recipients [161,162].

Ribavirin alone or in combination with pegylated interferon-alpha can also be utilized in non-transplant immunosuppressed patients with HIV infection or hematological abnormalities in cases of chronic HEV infection [163,164,165]. Sofosbuvir is an NS5B polymerase inhibitor, approved by the Food and Drug Administration for the treatment of hepatitis C. Due to its in vitro efficacy, it was suggested as an option in the treatment of ribavirin-resistant HEV infections [166] in combination or not with ribavirin, with contrasting results [138].

When ribavirin-resistant or relapsing HEV infections exhibit markedly low blood zinc values, zinc has been proposed as an adjuvant treatment. However, more extensive research is required to comprehend the impact of adjuvant zinc therapy on individuals with persistent HEV infection who do not respond well to ribavirin treatment [138].

Under controlled laboratory settings, the natural substance silvestrol inhibited HEV multiplication [167]

It has never, however, been put to the test on people. GPC-N114 and NITD008 are used to treat picornavirus and dengue fever, respectively. These two medications were able to prevent HEV multiplication in test settings without causing appreciable cytotoxicity in cell cultures [168].

However, their safety and antiviral effectiveness for human HEV infections are still unclear. Eventually, in a phase 3 trial in China in 2010, a HEV vaccine based on a protein transcribed by ORF2 of HVE1 was tested. Since ORF2 encodes the capside protein, which contains neutralizing epitopes that cause the host and reservoirs to produce antibodies, it is a structural protein that may be targeted to create a viable vaccine [143].

The effectiveness and safety of Hecolin, the first vaccine made in China, which has not yet been given the go-ahead for marketing, need to be further investigated. In a phase 3 clinical study, this vaccine (30 mcg of pure recombinant hepatitis E antigen per dose) administered at 0, 1, and 6 months demonstrated 100% efficacy [67]. Before the vaccine is widely used, it must be proven safe and effective in individuals with chronic liver disease as well as in other groups, such as immunocompromised people.

Novel therapy strategies for HEV in patients with immunosuppressive conditions are clinically necessary. In spite of a significant risk of graft rejection, the research by Kamar et al. showed that lowering immunosuppressive treatment that particularly targets T cells might encourage HEV eradication in transplant recipients [169,170]. For solid-organ transplant recipients with HEV, the first-line therapy approach is to reduce the amount of immunosuppressive medicines, especially those that target T cells; mycophenolate mofetil appears to be able to prevent the rejection and decrease viral replication.

Large-scale research is necessary to fully comprehend the effects of some promising substances, like silvestrol, which has not been tested on humans but decreased fecal HEV-RNA in mice; zinc, which may serve as an adjuvant therapy in ribavirin-resistant or relapsed HEV infections; and 2′-C-methylguanosine, which inhibits the growth of HEV in cell cultures.

## 10. Considerations on the Role of Wild Boars as Source of Infectious Disease

In populations of wild boar, antibodies to several zoonotic viruses, such as the Japanese encephalitis virus, swine influenza virus, and hepatitis E virus (HEV), have been found [171]. These zoonotic viruses can spread from wild boars to people when they come into close contact with them. The Eurasian wild boar, or *Sus scrofa*, is found throughout most of Europe. Over the past 50 years, hunting bags, or the total number of wild boar that have been hunted, have shown a significant increase in the population, with over 2.2 million wild boar being harvested annually on the continent [172]. Because farmed pigs and wild boars are members of the same species (*Sus scrofa*), they can spread disease to one another. Therefore, diseased wild boar populations might pose a risk to global trade as well as the pig business [173]. African and classical swine fever, as well as swine brucellosis, are examples of extremely infectious viral infections that have been known to spread from wild boar to pigs. Given that wild pigs are known to transmit bovine tubercolosis [174], they can potentially infect household pets, other animals, and cattle. For instance, after coming into touch with wild boar, hunting dogs and wild predators have perished from infection with the Aujeszky’s disease virus. Finally, according to Meng and Lindsay (2009) [171], wild boar may harbor pathogens that affect humans, such as the hepatitis E virus [175], *Leptospira* sp. [176], *Trichinella* sp. [177], and bacteria that are known to cause foodborne illnesses [178]. Contacts between outdoor domestic pigs and free-ranging wild boar have been observed; these interactions might be viewed as a proxy for the risk of disease transfer. The kind of interaction where disease transmission is most likely to occur is hybridization. Growing research on the prevalence of pathogens across different nations indicates that hybridization in wild boar and pig populations increases the risk to human health. The following were found to be risk factors for contact: a distance of more than five meters between pig enclosures and piggery buildings; a distance of more than 500 m between pig enclosures and other residences; close to a forest (less than 500 m); electric fences; and fences that are shorter than sixty centimeters [179]. According to Wu et al. (2012) [180], pig farms with grassland generally had a higher risk than those with concrete ground. For the majority of illnesses that affect animals, there are a few common risk factors. A significant risk factor for wildlife illnesses is an abundance of animals [181]. There is risk associated with open-air farming as well. Because they are raised in greater quantities than other livestock species, some of them are more likely to transmit illnesses to wildlife [182]. Game birds, ducks, geese, and farmed deer are all included in this [183]. Concerns about animal welfare have also led to an increase in the conventional indoor-raised chicken and pig population in recent years, which raises the danger of disease transmission. Therefore, global veterinary health scenario implementation is required, utilizing a variety of eradication and control programs, close monitoring, and early warning systems. There are three main approaches to managing diseases in wildlife: preventing new infections from spreading, controlling diseases that are already present, or attempting a virtually unachievable eradication [184]. Using interdisciplinary teams to integrate veterinary, ecological, and wildlife management knowledge is highly advised in all cases [185]. Controlling wildlife illnesses starts with monitoring, which involves identifying the diseases that are there, their historical and current distribution, and any patterns in their occurrence. In Europe, a number of regional, national, and international programs have addressed wildlife disease surveillance. At the moment, certain regions of Europe receive the benefits of wildlife surveillance (usually restricted to a few illnesses), whereas other regions receive no surveillance at all. It is acknowledged that countries that conduct disease surveillance of their wild animal populations are more likely to identify the presence of infectious and zoonotic diseases and to quickly adopt countermeasures, so the veterinary authorities must prioritize the proper implementation of a comprehensive surveillance effort [186]. To limit the spread of epidemic diseases, several approaches can be implemented. It is rare to erect barriers to keep domestic cattle and wildlife apart, and when it does, it is only done in high-risk circumstances. For instance, in tuberculosis endemic areas, it could be wise to stay away from badgers on cattle farms or to keep wild boar away from open-air raised pigs or other animals [187]. Every nation and hunting method should be required to follow hygienic procedures, such as properly disposing of hunting carcasses and carcass remnants [188]. To preserve some animal species, which often only exist in extremely small numbers and are on the brink of extinction in Europe, further study is necessary to determine the actual hazards associated with such management in each unique circumstance. Eliminating animals is nearly never a successful strategy for managing diseases affecting wildlife. Intense scientific and social discussion surrounds it as well (for example, on badger culling as a means of controlling tuberculosis) [189]. Culling and eradication are only options when dealing with point-source wildlife disease outbreaks (focusing culling on the disease focus plus an outer ring of vaccination) or island populations, where geographical barriers limit animal dispersal, or introduced species (pest species, where legal and social constraints to culling are minimal). In contrast, many disease control initiatives aim to reduce the population. This is a stopgap approach, until habitat alteration is employed to modify host distribution or exposure to disease agents, or to more permanently lower host density [184,190].

## 11. Conclusions

HEV poses a significant global health threat, requiring accurate diagnosis and comprehensive public health interventions for effective control. A combined approach utilizing serological screening followed by molecular confirmation optimizes resource allocation. Standardized sampling, testing, and data interpretation are essential for generating reliable and comparable results across studies and regions. Continued research towards more efficient, cost-effective, and field-friendly diagnostic tools is vital for improved surveillance and control efforts. From this perspective, implementing biomarker research suitable for the unbiased diagnosis might indicate the way. These might be effectively implemented into cutting-edge technologies such as biosensors, electronic noses, and lateral flow tests to hamper public and autonomous monitoring. Robust surveillance systems, including timely reporting, molecular surveillance, and monitoring HEV prevalence in animal reservoirs, such as the wild boar, are critical for guiding effective public health interventions. International collaboration is key for sharing information and coordinating control efforts. Finally, addressing health disparities by ensuring equitable access to clean water, sanitation, healthcare, and health education, particularly in low-resource settings, is paramount. Public health interventions must be tailored to the specific needs and challenges of diverse populations. In conclusion, combating HEV requires a comprehensive strategy encompassing accurate diagnosis, robust public health interventions, and ongoing research. By prioritizing these efforts, we can significantly reduce the global burden of HEV and safeguard public health using a robust One-Health model.

## Figures and Tables

**Table 1:** Epidemiological patterns of HEV.

Characteristics	Low Endemic	Large Endemic
Transmission pathways	via blood transfusions and animal sources	Mostly via blood transfusions and contaminated water sources
Frequently observed genotypes	3 and 4	1 and 2
Symptoms	Acute hepatitis with and without symptoms. Immunocompromised people with chronic hepatitis	Frequent outbreaks, asymptomatic and symptomatic acute hepatitis, acute liver failure
Countries involved	Europe, North and South America, South Africa, Australia, and East Asia	Central America, Africa, South and Central Asia

**Table 2:** Prevention measures.

Interventions on Exposure Routes	Interventions on Categories of People	Infected People
Increasing the amount and quality of drinking water	Prenatal examinations for pregnant women	Prompt case management and diagnosis
Correct handling and disposal of human waste		Prompt recommendation for medical care
Enhancing personal hygiene		Steer clear of giving needless hepatotoxic medications
Cooking wholesome, safe food		
